# Supplementary material for: Trends in proton pump inhibitor use, reflux esophagitis, and various upper gastrointestinal symptoms from 2010 to 2019 in Japan
Source: PLoS One. 2022 Jun 17;17(6):e0270252. doi: 10.1371/journal.pone.0270252 (PMC9205527; doi:10.1371/journal.pone.0270252)
Supplement: Attachment — Submitted filename: Rebuttal 01_PLOSONE.doc [file pone.0270252.s002.doc]

Dear Editor of *PLoS ONE*

We are grateful to resubmit our manuscript (PONE-D-22-02306) entitled “Trends in proton pump inhibitor use, reflux esophagitis, and various upper gastrointestinal symptoms from 2010 to 2019 in Japan”. According to the editor and reviewers’ instructions, we revised our manuscript as follows.

Reviewer #1-1): The tables appear to be overly convoluted and difficult to read, mostly because of an abundance of duplicate entries from the FSSG questionnaire (Heartburn, heartburn while bending, heartburn after meals). I wonder if a simplification of the symptoms categories would be possible including clustering similar symptoms together (heartburn) and/or removing symptoms which are not very specific to GERD (feeling sick after meals) as tables are very difficult to read in their present form. Perhaps splitting symptoms into typical (HB, regurgitation, chest pain and dysphagia) and atypical could help. All the 12 entries could be left as is in table 5.

Thank you very much for this keen comment. We think the detailed data of 12 upper GI symptoms of FSSG is important and useful for expert doctors of this field. FSSG has been actually and widely used in Japan for more than 15 years. However, we agree that it is rather complicated and difficult for readers to understand.

According to the advice, we have merged the 12 upper GI symptoms into three categories; GERD-related gastrointestinal symptoms (heartburn or acid regurgitation), GERD-unrelated symptoms (dyspepsia or fullness while eating), and other symptoms of pharyngo-upper esophageal area (pharyngo-upper esophageal discomfort). A simplified table is newly added as Fig. 5c, which makes readers understand our results more easily.

To explain the newly added figures, we added the description in the Result Section (page 10, line 169 to line 177) and Discussion section (page 11, line 207 to page 12, line 216) in the revised manuscript (the file name is revision_Final_off_without TrackChange.docx).

Reviewer #1-2): It would be interesting to know the schedule and indications for EGD surveillance and whether the authors subscribe to ASGE guidelines

Thank you very much for this comment. We agree the schedule for EGD surveillance is quite important. About the 217,712 subjects who were evaluated in our analyses, the number of times they underwent EGD during the 10 years ranged from 1 to 17 (a graph shown below).

However, we think this graph is not useful for readers worldwide, because the major reason for EGD screening varies by countries. In most cases in Japan, EGD surveillance is performed to detect gastric cancer, not to check the status of reflux disease or Barrett’s esophagus. Due to the high prevalence of *Helicobacter pylori* infection, gastric cancer is one of the most severe clinical problems in Japan, which is quite different from the situation of United States or other Western countries. Therefore, it is difficult to apply our data to the guideline of ASGE or those of other Western countries.

Reviewer #1-3): It seems like the most common symptom the patient report is bloating, which is not usually improved by PPIs administration. There is however an improvement in patients reporting this symptom amongst PPI takers. This should be expanded upon in the discussion.

We appreciate this fruitful comment. According to this advice, we added the new paragraph in the Discussion section (page 11, line 204 to page 12, line 216) in the revised manuscript (the file name is revision_Final_off_without TrackChange.docx). It is certain that some upper GI symptoms such as dyspepsia, bloating, etc. are generally considered as the symptoms with little relation to gastric acid. But our result and the recent meta-analysis (reference #37 newly cited in the revision) suggest that PPI may be effective to such symptoms to some extent.

Reviewer #1-4): It would be interesting to know how many of the patients underwent anti-reflux surgery for their symptoms. I know its not very popular in Japan but perhaps some of the patients underwent this route.

Thank you for the comment. As the reviewer commented, the number of people who undergo anti-reflux surgery is very few in Japan. Our study population comprised of normal healthy people, and no one underwent anti-reflux surgery from 2010 to 2019.

On the contrary, about one hundred participants developed gastric cancer and underwent endoscopic or surgical treatment during the study period (from 2010 to 2019), probably due to the high prevalence of *H. pylori* infection in Japan (this result is now in preparation for another journal).

Reviewer #1-5): Each figure should have a description for easier interpretability

We are quite sorry that our figure legends were difficult to understand. According to the advice, we have changed the legends of Figure 1, Figure 3, Figure 4, and Figure 5 in more detail for readers to understand our results easily.

Reviewer #1-6): given the detail of the database it would be good to know if authors also collected other medical history hinting to PPIs side effects such as PNA, C. Difficlile Colitis, fractures and esophageal cancer rates.

Thanks for the important advice, and we completely agree that PPI side effects is a very important clinical point. However, at present we do not have the data of PNA, C. Difficile colitis, fractures, or esophageal cancer. We will evaluate the relation of PPI use and such diseases in our future analyses.
